# Tungstate-Targeting of BKαβ_1_ Channels Tunes ERK Phosphorylation and Cell Proliferation in Human Vascular Smooth Muscle

**DOI:** 10.1371/journal.pone.0118148

**Published:** 2015-02-06

**Authors:** Ana Isabel Fernández-Mariño, Pilar Cidad, Delia Zafra, Laura Nocito, Jorge Domínguez, Aida Oliván-Viguera, Ralf Köhler, José R. López-López, María Teresa Pérez-García, Miguel Ángel Valverde, Joan J. Guinovart, José M. Fernández-Fernández

**Affiliations:** 1 Laboratori de Fisiologia Molecular i Canalopaties, Departament de Ciències Experimentals i de la Salut, Universitat Pompeu Fabra, Barcelona, Spain; 2 Departamento de Bioquímica y Biología Molecular y Fisiología and Instituto de Biología y Genética Molecular (IBGM), Universidad de Valladolid and Consejo Superior de Investigaciones Científicas (CSIC), Valladolid, Spain; 3 Institute for Research in Biomedicine (IRB Barcelona) and Department of Biochemistry and Molecular Biology, University of Barcelona, and Centro de Investigación Biomédica en Red de Diabetes y Enfermedades Metabólicas (CIBERDEM), Barcelona, Spain; 4 Aragon Institute of Health Sciences I+CS/IIS and Fundación Agencia Aragonesa para la Investigación y Desarrollo (ARAID), Zaragoza, Spain; Universidad de La Laguna, SPAIN

## Abstract

Despite the substantial knowledge on the antidiabetic, antiobesity and antihypertensive actions of tungstate, information on its primary target/s is scarce. Tungstate activates both the ERK1/2 pathway and the vascular voltage- and Ca^2+^-dependent large-conductance BKαβ_1_ potassium channel, which modulates vascular smooth muscle cell (VSMC) proliferation and function, respectively. Here, we have assessed the possible involvement of BKαβ_1_ channels in the tungstate-induced ERK phosphorylation and its relevance for VSMC proliferation. Western blot analysis in HEK cell lines showed that expression of vascular BKαβ_1_ channels potentiates the tungstate-induced ERK1/2 phosphorylation in a G_i/o_ protein-dependent manner. Tungstate activated BKαβ_1_ channels upstream of G proteins as channel activation was not altered by the inhibition of G proteins with GDPβS or pertussis toxin. Moreover, analysis of G_i/o_ protein activation measuring the FRET among heterologously expressed G_i_ protein subunits suggested that tungstate-targeting of BKαβ_1_ channels promotes G protein activation. Single channel recordings on VSMCs from wild-type and β_1_-knockout mice indicated that the presence of the regulatory β_1_ subunit was essential for the tungstate-mediated activation of BK channels in VSMCs. Moreover, the specific BK channel blocker iberiotoxin lowered tungstate-induced ERK phosphorylation by 55% and partially reverted (by 51%) the tungstate-produced reduction of platelet-derived growth factor (PDGF)-induced proliferation in human VSMCs. Our observations indicate that tungstate-targeting of BKαβ_1_ channels promotes activation of PTX-sensitive G_i_ proteins to enhance the tungstate-induced phosphorylation of ERK, and inhibits PDGF-stimulated cell proliferation in human vascular smooth muscle.

## Introduction

Tungstate has antidiabetic and antiobesity actions in several animal models: 1) tungstate treatment normalizes hepatic carbohydrate metabolism [1, 2]; 2) stimulates insulin secretion and regenerates pancreatic β-cell population [3]; 3) mimics the effect of insulin on hepatocytes (but in an insulin receptor independent manner) by increasing glycogen synthesis and deposition [4]; 4) increases the production and translocation of the insulin-regulated glucose transporter GLUT4 in muscle [5]; 5) favors thermogenesis and lipid oxidation in adipose tissue [6]; and 6) modulates hypothalamic gene expression by activation of the leptin-signaling pathway responsible for the regulation of food intake and energy expenditure [7]. In addition, tungstate also reduces blood pressure in experimental animal models of both hypertension [8, 9] and metabolic syndrome [10].

Despite this knowledge on tungstate effects, our understanding of the underlying molecular mechanisms is incomplete. In this respect, it has been suggested that activation of several kinases (extracellular signal-regulated kinases (ERK) 1/2 and JAK2) by tungstate can lead to some of its antidiabetic and antiobesity actions [4, 5, 7]. Indeed, tungstate stimulates ERK phosphorylation in different cell types, including CHO cells, Leydig cells, neurons, and hepatocytes, leading to the phosphorylation (inactivation) of the glycogen synthase kinase-3β that in turn modulates cell function [4, 11, 12]. Although the nature of tungstate targets upstream of ERK phosphorylation is not fully known, the involvement of a non-canonical pathway that requires pertussis toxin (PTX)-sensitive G_i/o_ proteins has been proposed recently, at least in CHO and liver cells [13].

Tungstate’s antihypertensive actions seem to be achieved by inhibition of endothelial xanthine oxidase [8] and by activation of the large-conductance voltage- and Ca^2+^-activated K^+^ (BK) channel in vascular smooth muscle [14]. These BK channels are mostly formed by tetramers of the pore-forming α subunit (encoded by a single gene, *KCNMA1*) along with the regulatory β_1_ subunit (encoded by the *KCNMB1* gene). This accessory β_1_ subunit favors BK channel activation by voltage and Ca^2+^ [15–17]. BK channels are pivotal in the regulation of arterial tone, where they facilitate a negative feedback mechanism which opposes the vasoconstriction driven by Ca^2+^ entry through voltage-gated L-type Ca^2+^ channels. Activation of L-type Ca^2+^ channels by membrane depolarization not only leads to vascular smooth muscle contraction but also promotes the opening of ryanodine receptors in the sarcoplasmic reticulum. BK channels are activated in vascular smooth muscle by local and transient Ca^2+^ increases (“Ca^2+^ sparks”) caused by the opening of a cluster of ryanodine receptors in the sarcoplasmic reticulum membrane adjacent to the cell membrane. The efflux of K^+^ through BK channels is sufficient to hyperpolarize the membrane potential limiting membrane depolarization, Ca^2+^ influx via voltage-operated L-type Ca^2+^ channels, and smooth muscle contraction [18]. Because of its action on BK channel function, the presence of the regulatory β_1_ promotes this negative impact on vascular resistance [19, 20]. Moreover, the presence of β β_1_ is required for channel modulation by a series compounds [21–25], in particular, tungstate. Still, the putative binding site of tungstate has been mapped to the pore-forming α channel subunit, in close proximity to the Mg^2+^ binding site [14, 26].

It has been suggested that K^+^ channels serve as upstream modulators of the ERK pathway [27, 28]. As a general biophysical principle, cell membrane hyperpolarization caused by K^+^ channel activation increases the driving force for Ca^2+^ entry, and the resulting increase in intracellular Ca^2+^ concentration enhances cell proliferation by stimulating Ca^2+^-sensitive signaling steps. However, additional roles of K^+^ channels as direct transducers of intracellular signals, beyond their ion-conducting function and hyperpolarizing action, are emerging [29]. For example, it has been suggested that the intermediate-conductance Ca^2+^-dependent K^+^ (IK1) channel (also known as K_Ca_3.1 or SK4, and encoded by the *KCNN4* gene) can promote cell proliferation independent of K^+^ conductance, by direct interaction with ERK1/2 and JNK signaling pathways [30].

Phenotypic modulation of vascular smooth muscle cells (VSMCs) from a contractile phenotype toward a proliferative phenotype and concomitant vascular remodeling is a hallmark of vascular pathologies such as hypertension, hyperlipidemia or neointima formation after balloon catheter intervention [31, 32]. At the molecular level, such phenotypic remodeling has been linked to changes in the expression of many genes including K^+^ channels such as K_Ca_3.1 [32] and voltage-gated K^+^ channels belonging to the K_V_1/5 subfamilies [33, 34]. Although the activation of ERK1/2 has been involved in the proliferation of VSMCs induced by growth factors such as platelet-derived growth factor (PDGF) [35], dedifferentiated VSMCs gradually regain contractile functions in a process mediated by PTX-sensitive G proteins (in particular, Gβγ dimmers) that also relies on ERK pathway activation [36, 37]. Such controversial role of the ERK pathway in VSMC phenotypic modulation can be explained at least in part by differences in both strength and duration of ERK1/2 phosphorylation [34, 35].

Given that tungstate promotes the activity of both the ERK1/2 pathway (in a G_i/o_ protein-dependent manner) and the BKαβ_1_ channel, we hypothesized and found that BK channel plays a mechanistic regulatory role in the tungstate-induced ERK phosphorylation that is relevant for VSMC proliferation.

## Materials and Methods

### Reagents

Sodium tungstate, Pertussis Toxin (PTX), GDPβS, Noradrenaline (NA) and EGF were from Sigma-Aldrich. Iberiotoxin (IbTX) was from Alomone Labs Ltd. (Jerusalem, Israel). Tissue culture media and supplements were from Sigma and Invitrogen. Fetal bovine serum (FBS) was from Gibco. Phospho-ERK antibody (1:1,000 in BSA blocking solution) was purchased from Cell Signaling TECHNOLOGY. Monoclonal mouse anti-β-Actin 1:10,000 (ab8226, Abcam, Cambridge, UK), was used in vascular smooth muscle cells (VSMCs) Western blots as loading control. For PTX experiments, transfected cells were incubated with 100–500 ng/ml PTX in culture medium, at 37°C and for 24–28 hours before electrophysiological recording or Western blot assays.

### cDNA constructs

Human β_1_ subunit (KCNMB1) of the BK channel (cloned into pcDNA3) was a gift from Dr. Ligia Toro (University of California—Los Angeles, Los Angeles, California, USA). Human α subunit (KCNMA1) of the BK channel (cloned into pcDNA3) was supplied by Dr. Ramón Latorre, (Centro de Neurociencias de Valparaíso, Valparaíso, Chile). Rat Gα subunit (tagged with YFP), human Gβ subunit (tagged with CFP) and adrenergic α_2A_ receptor (α_2A_-AR) (all cloned into pcDNA3) were kindly supplied by Dr. Moritz Büneman (Department of Pharmacology and Toxicology, University of Würzburg—Germany).

### Human VSMCs collection and culture

Human renal arteries belonging to the COLMAH collection of the HERACLES network (http://www.redheracles.net/plataformas/en_coleccion-muestras-arteriales-humanas.html) were obtained from donors at the Clinic Hospitals of Barcelona and Valladolid. Vessels were placed in a Dulbecco’s modified Eagle’s medium (DMEM), for cell isolation. Samples were received within 24 hours after intervention and kept at 4°C. Cultured vascular smooth muscle cells (VSMCs) were obtained from cell outgrowth of vessels explant as described elsewhere [33]. Briefly, VSMCs were isolated from the medial layer of the vessel kept in DMEM after manual removal of both adventitia and endothelial layers under a dissection microscope. Once cleaned, the muscle layer was cut in 1 mm^2^ pieces that were seeded in 35 mm Petri dishes treated with 2% gelatin (Type B from bovine skin, Sigma) or collagen (6 well multidish collagen, Thermo Scientific), in DMEM supplemented with 20% FBS, penicillin-streptomycin (100 U/ml each), 5 μg/ml fungizone, and 2 mM L-glutamine (Lonza) at 37°C in a 5% CO_2_ humidified atmosphere. Migration and proliferation of VSMCs from the explants were evident within 10–15 days. Confluent cells were trypsinized and seeded at 1/3 density and VSMCs were subjected to several (up to 8) passages in control DMEM supplemented with 5% FBS, penicillin-streptomycin (100 U/ml each), 5 μg/ml fungizone, 2 mM L-glutamine, 5 μg/ml Insulin, 1 ng/ml bFGF and 5 ng/ml EGF.

### Transfection of HEK293 cell lines

For electrophysiological analysis, HEK293 cells were transfected using polyethylenimine ExGen 500 (Fermentas Inc., Hanover, MD, USA), following the manufacturer’s instructions [seven equivalents of polyethylenimine per 3.3 μg of cDNAs (cloned into pcDNA3 vector) expressing the human BK α subunit together with the human β_1_ subunit (1:2 ratio) and the transfection reporter pEGFPN1].

For FRET studies, HEK293 cells, HEKα cells (expressing constitutively the human α subunit of the BK channel) and HEKαβ_1_ cells (constitutively expressing the bovine α and β_1_ subunit of the BK channel) were transiently transfected with rat Gα-YFP (YFP inserted between position 91 and 92), human CFP-Gβ fusion protein (CFP fused to the N-terminus) and the Gγ subunit using polyethylenimine ExGen 500 (Fermentas Inc., Hanover, MD, USA). For control FRET experiments, the cDNA corresponding to α_2A_-AR was also co-expressed in HEK293 cells.

### Western blot analysis

The different HEK293 cell lines were grown to confluence and then deprived of FBS overnight. For the study of ERK1/2 phosphorylation in human renal VSMCs, 20,000 cells were seeded on 12 mm diameter plates in control medium and after 24 hours, cells were made quiescent with serum-free medium for 48 hours. The different treatments (Tungstate, PDGF, IGF, EGF or tungstate + IbTX) were performed in serum-free medium. Plates were flash frozen in liquid nitrogen and processed for protein extract preparation. Protein concentration was measured using the BCA Protein Assay (Pierce, USA). Proteins were separated by SDS—PAGE loading 20 μg of total protein per lane, transferred to nitrocellulose membranes (Schleicher and Schuell, Dassel, Germany) and immunoblotted with selected antibodies. Proteins were visualized using an enhanced chemiluminescence detection system (GE Healthcare, UK) or with the VersaDoc 4000 Image System (BioRad) with chemiluminescence reagents (SuperSignal West Femto Chemiluminescent Substrate, Pierce Biotechnology). The relative amount of protein was calculated by densitometric analysis of bands and, in the case of VSMCs, normalized to their corresponding β-actin signals using the Fiji (Image J) software.

### Electrophysiology

Equation 1 Inside-out BK currents were recorded in macropatches from EGFP-positive HEK cells 2–3 days after transfection. Borosilicate glass patch pipettes had a tip resistance of 2–3 MΩ and were filled with a solution containing (in mM): 140 KCl, 1.2 MgCl_2_, 0.15 CaCl_2_, 5 EGTA, 10 HEPES (300 mOsm/l, pH 7.35). 0 Ca^2+^ solution (nominal 0 Ca^2+^) bathing the cytoplasmic face of the membrane patch, to which 1 mM WO_4_^2-^ was added for 5–10 minutes after a series of control recordings, contained (in mM): 140 KCl, 0.7 Mg^2+^, 5 EGTA, 10 HEPES (300 mOsm/l and pH 7.25). We compensated series resistance to 80–90% because of the large magnitude of BK currents. When used, GDPβS was added to the 0 Ca^2+^ bath solution 5 minutes before starting the electrophysiological experiments. For current-activation studies, membrane macropatches were clamped at 0 mV, pulsed for 150 milliseconds from—100 mV to +200 mV in 10 mV steps, and repolarized to—80 mV for 20 milliseconds. Experiments were performed at room temperature (22–26°C). Relative conductance was determined by measuring tail current amplitudes at—80 mV. For each patch the conductance-voltage (G-V) relationship was fitted with the Boltzmann equation:

$$\frac{\text{G}}{\text{Gmax}}=\frac{1}{1+\text{exp}(-\frac{\text{V}-\text{V}1/2\text{ act}}{\text{kact}})}$$

where G is the value of the instantaneous tail current at each test voltage, Gmax is the maximum obtained tail current, V is the test voltage applied to the membrane, V_1/2 act_ is the voltage for half-maximal current activation, and k_act_ (an index of the minimum number of elementary charges that move through the electric field to gate the channel) is the slope factor of the Boltzmann term. V_1/2 act_ is a convenient parameter to study the effect of BK channel modulators since it is directly related to the energy required to open the channel. Single G-V curves from a set of patches at each experimental condition were averaged to obtain the shown G-V curves.

For single channel recordings, myocytes were obtained by enzymatic digestion after surgical extraction of the aortic artery from previously euthanatized +/+ (wild-type) and −/− BKβ_1_ (β_1_-knockout) C57BL/6 mice (sacrificed by cervical dislocation). Aortic arteries were digested enzymatically with a mixture of papain, bovine serum albumin (BSA) and collagenase (from Sigma) solved in Hank solution containing (in mM): 125 NaCl, 5.36 KCl, 5 NaHCO_3_, 0.34 NaHPO4, 0.44 KHPO_4_, 10 glucose, 1.45 sucrose and 10 HEPES (pH adjusted to 7.4 with NaOH 10N). After mechanical dispersion in 100 nM Ca^2+^ recording solution, containing (in mM): 140 KCl, 5 EGTA, 10 HEPES, 0.7 MgCl_2_, 2.4 CaCl_2_, (pH adjusted to 7.2–7.3 with TRIS-BASE and 300 mOsm/l), vascular myocytes were seeded in plastic dishes (35 mm). Electrophysiological studies were performed in the first 8 hours after the extraction of arteries. Single-channel currents were recorded from inside-out macropatches clamped at 0 mV, pulsed for 30 seconds at different depolarizing voltages using a gap-free protocol. Borosilicate glass patch pipettes had 2–3 MΩ of resistance and were filled with a solution containing (in mM): 140 KCl, 1.2 MgCl_2_, 5 EGTA, 10 HEPES (300 mOsm/l, pH 7.35). All experiments were performed at room temperature with the 100 nM Ca^2+^ free recording bath solution. Tungstate (1 mM) was added to the bath solution with a fast perfusion system after a series of control recordings.

Measurements of K_Ca_3.1 currents in 3T3 murine fibroblasts were performed as described previously [38]. In brief, membrane currents were recorded in the standard whole-cell mode by using an EPC10-USB patch-clamp amplifier (HEKA, Germany) and voltage ramps ranging from-100 mV to +100 mV (duration 1 sec). The KCl-pipette solution-suitable as intracellular solution for K_Ca_ channel recording and activation- contained (in mM): 140 KCl, 1 MgCl_2_, 2 EGTA, 1.71 CaCl_2_ (1 μM [Ca^2+^]_free_) and 5 HEPES (adjusted to pH 7.2 with KOH). The bath solution contained (in mM): 140 NaCl, 5 KCl, 1 MgCl_2_, 1 CaCl_2_, 10 glucose, and 10 HEPES (pH 7.4).

The pClamp8, PatchMaster and FitMaster softwares were used for pulse generation, data acquisition, and subsequent analysis. Currents were recorded at 10 kHz and low-pass-filtered at 1 kHz.

### FRET experiments

Ratiometric FRET was obtained by excitation (458 nm) of the CFP-Gβ CFP and emission (514 nm) of Gα-YFP YFP in a Leica TCS SP5 confocal microscope equipped with a 63x Oil objective and analyzed using the ImageJ software. R0 (the distance at which 50% energy transfer takes place) for the donor-acceptor CFP/YFP FRET pair has been reported to be 4.92 nm [39]. All FRET experiments were carried at room temperature and the cells were bathed in a solution containing (in mM): 140 NaCl, 1 MgCl_2_, 1.2 CaCl2, 10 HEPES, 5 glucose, 0.5 EGTA (300 mOsm/l and pH 7.2–7.3). Noradrenaline (NA) was added to this bath solution in order to determine agonist-induced changes in FRET from HEK293 cells transfected with cDNAs encoding Gα-YFP, CFP-Gβ, Gγ and α_2A_-AR.

### Proliferation assays

Human renal VSMCs at passages 3–8 were seeded onto poly-l-lysine coated coverslips placed in 12 mm wells at a density of 20,000 cells/well. Cells were maintained in control medium for 24 hours and synchronized in serum free (SF) medium during 48 hours. Then, PDGF (20 ng/ml) was added for an additional 24 hours period. Tungstate and/or the BK channel blocker IbTX were added one hour before and remained present during PDGF stimulation. The percentage of cells at the S phase was quantified using EdU (5-ethynyl-2´-deoxyuridine) incorporation for another 6 hours with a commercial kit (Click-iT EdU Imaging Cell Proliferation Assay, Invitrogen). Finally, cells were incubated with Hoechst before mounting with Vectashield (Vector Laboratories Inc.,Burlingame, CA). EdU incorporation was visualized with an immunofluorescence microscopy (Nikon) at the corresponding wavelength depending on the Alexa Fluor used and was expressed as the percentage of the total cell number stained with Hoechst. In each experiment, this percentage was an average of 10 to 20 high power fields per coverslip. Triplicates were made for each condition.

### Ethics Statement

Protocols to obtain human renal arteries from donors at the Clinic Hospitals of Barcelona and Valladolid, were approved by the Human Investigation Ethics Committee of the Hospitals. These samples belong to the COLMAH collection of the HERACLES network (http://www.redheracles.net/plataformas/en_coleccion-muestras-arteriales-humanas.html).

Murine aortic arteries were obtained from mice sacrificed by cervical dislocation. Animal protocols for *in-vitro* studies were performed in agreement with ARRIVE guidelines and the Spanish legislation on protection of animals, specifically approved by the local Institutional Animal Care and Use Committee (Comité Ético de Experimentación Animal del Consorci Parc de Recerca Biomèdica de Barcelona, including Universitat Pompeu Fabra) (Approval ID: JMC-07–1001P2) and conformed to the Directive 2010/63/EU of the European Parliament.

### Statistics

Data are presented as means ± S.E.M. For statistical comparison of two and more groups we used the Student’s *t* test, Mann-Whitney U-test, One Way Analysis of Variance (ANOVA) followed by Tukey *post hoc* test, or Nonparametric ANOVA (Kruskal-Wallis Test) followed by Dunn *post hoc* test, where appropriate. Differences were considered significant if P < 0.05.

## Results

### BKαβ_1_ channels play a role in the G_i/o_ protein-dependent ERK1/2 phosphorylation induced by tungstate

**Fig 1:**
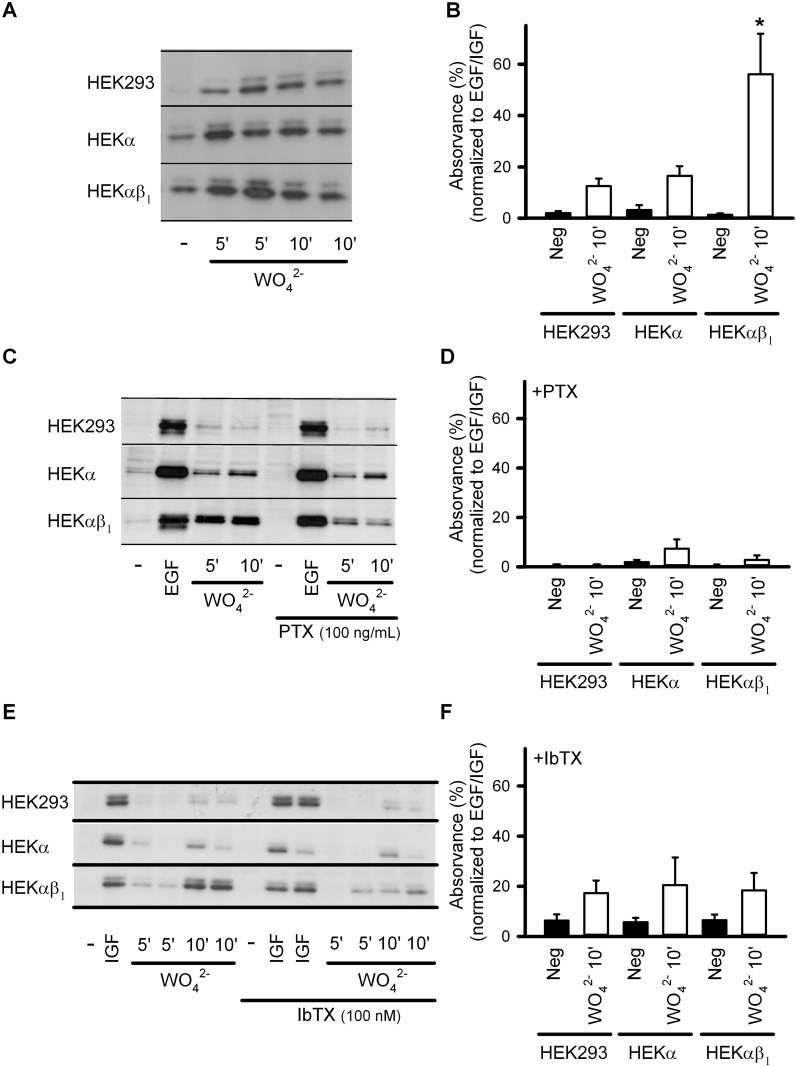
BKαβ_1_ channels potentiate tungstate-induced ERK1/2 phosphorylation in a G_i/o_ protein-dependent manner. Phosphorylation of ERK1/2 was analyzed by Western blot using phospho-ERK-specific antibodies. Total ERK was used as loading control (data not shown). Protein expression and phosphorylation were quantified by densitometry of the corresponding Western blot signal. Relative density (phosphorylated versus total ERK) was normalized to the inner control (EGF/IGF for each condition, which was considered as 100%). (A) Representative Western blots obtained from HEK293, HEKα and HEKαβ_1_ cells for ERK1/2 phosphorylation levels, without treatment (-) or after treatment with 1 mM tungstate (WO_4_^2-^) (during 5 and 10 minutes, as indicated) in the absence of toxins. (B) Average normalized relative density (phosphorylated versus total ERK) in the absence of toxins. (C) Representative Western blots obtained from HEK293, HEKα and HEKαβ_1_ cells for ERK1/2 phosphorylation levels, without treatment (-), after treatment with 100 ng/ml EGF (during 10 minutes) (EGF) or after treatment with 1 mM tungstate (WO_4_^2-^) (during 5 and 10 minutes, as indicated) in the absence (left) or presence of PTX (right). (D) Average normalized relative density (phosphorylated versus total ERK) in the presence of PTX. (E) Representative Western blots obtained from HEK293, HEKα and HEKαβ_1_ cells for ERK1/2 phosphorylation levels, without treatment (-), after treatment with 100 ng/ml IGF (during 10 minutes) (IGF) or after treatment with 1 mM tungstate (WO_4_^2-^) (during 5 and 10 minutes, as indicated) in the absence (left) or presence of IbTX (right). (F) Average normalized relative density (phosphorylated versus total ERK) in the presence of IbTX. n = 4–12 in each experimental group. *P < 0.05 when compared to the other HEK cell lines (Kruskal-Wallis test followed by Dunn *post hoc* test). See Methods for further details.

We have previously reported that tungstate only promoted the activation of heterologously expressed BK channels in the presence of regulatory β_1_ or β_4_ subunits [14]. In order to evaluate the possible role of BK channels in the tungstate-mediated activation of the ERK pathway, we analyzed ERK1/2 phosphorylation using phospho-specific antibodies. Western blot analysis of phosphorylated ERK1/2 was carried out in tungstate (1 mM)-treated non-transfected HEK293 cells, HEKα cells (constitutively expressing the human α subunit of the BK channel), and HEKαβ_1_ cells (constitutively expressing the bovine α and β_1_ subunits of the BK channel). Tungstate increased the phosphorylation of ERK1/2 in all HEK293 cell lines, although to a significantly higher level in HEKαβ_1_ cells (P < 0.001) (Fig. 1A, and 1B). Such enhanced ERK phosphorylation induced by tungstate in HEKαβ_1_ cells was prevented by pretreatment with either the G_i/o_ protein inhibitor pertussis toxin (PTX, 100 ng/ml) (Fig. 1C, 1D) or the specific BK channel blocker iberiotoxin (IbTX, 100 nM) (Fig. 1E, 1F). Both toxins were without effect on the EGF/IGF-induced ERK phosphorylation that we used as control for ERK activation.

### BKαβ_1_ channels function upstream of G_i/o_ protein activation induced by tungstate

**Fig 2:**
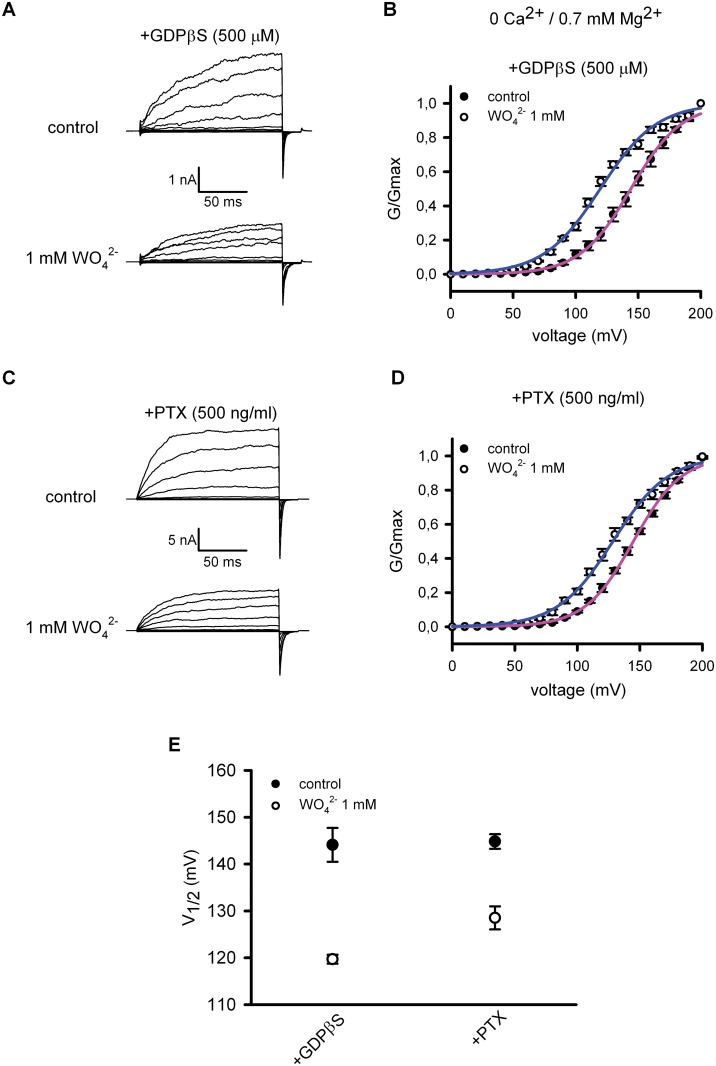
Tungstate-mediated activation of BKαβ_1_ channels is independent of G proteins. Representative currents recorded from excised inside-out macropatches obtained from HEK293 cells expressing the BKαβ_1_ channels in the presence of 500 μM GDPβS (added to the bath solution) (A) or from transfected HEK293 cells pre-incubated with PTX (500 ng/ml, 24 hours) (C). Currents were recorded at cytosolic 0 Ca^2+^ and 0.7 mM Mg^2+^ before (control, top panels) and 5–10 minutes after cytosolic application of 1 mM tungstate (WO_4_^2-^, bottom panel). The voltage protocol was as described in the Methods. (B), (D) Average G-V curves for BKαβ_1_ channels under the experimental conditions above mentioned. Solid curves were obtained by fitting the normalized conductance to the Boltzmann equation (see Methods). (E) Voltage for half maximal activation (V_1/2 act_) of BKαβ_1_ channels before (control, filled circles) and after addition of tungstate (1 mM WO_4_^2-^, open circles) obtained for the indicated experimental conditions (+GDPβS, n = 4; +PTX, n = 6). No significant difference was found in the decrease on V_1/2 act_ induced by tungstate when comparing both treatments: 24 ± 4 mV (n = 4) for GDPβS treatment *versus* 16 ± 3 mV (n = 6) for PTX treatment (P = 0.11, Mann-Whitney U-test). Besides the substantial difference in the duration of both treatments to inhibit G proteins (24–28 hours for PTX treatment and minutes for GDPβS treatment), no significant difference was found among them regarding V_1/2 act_ before the addition of tungstate (control situation for GDPβS treatment: V_1/2 act_ = 144 ± 4 mV (n = 4) *versus* control situation for PTX treatment: V_1/2 act_ = 145 ± 2 mV (n = 6); P > 0.99, Mann-Whitney U-test). Furthermore, these V_1/2 act_ control values (before tungstate application) were similar to the ones previously reported by us under identical experimental conditions but without interfering with the activation of G proteins: 139 ± 2 mV (n = 7) [14] and 147 ± 2 mV (n = 7) [26] (P = 0.1, ANOVA). Note that, as previously reported, 1 mM tungstate also reduced substantially the K^+^ current amplitude in the absence of cytosolic Ca^2+^ (A, C), an effect that, contrary to the tungstate-induced reduction of V_1/2 act_, has been shown to occur either in the absence or presence of Mg^2+^ and in the absence or presence of the different regulatory β subunits (β_1_-β_4_) [14]. For each experimental condition V_1/2 act_ and k_act_ values were (in mV): 144 ± 4 and 20 ± 0.3 (control situation for GDPβS treatment, n = 4); 120 ± 1 and 22 ± 1 (after WO_4_^2-^ addition for GDPβS treatment, n = 4); 145 ± 2 and 19 ± 0.3 (control situation for PTX, n = 6); 129 ± 2 and 21 ± 1 (after WO_4_^2-^ addition for PTX treatment, n = 6). No significant differences were found among k_act_ values (P = 0.07, ANOVA).

Since the BKαβ_1_ channel-dependent enhancement of tungstate-induced ERK phosphorylation was prevented by inhibition of G_i/o_ proteins, we next evaluated whether the BK channel operated either upstream or downstream of the G protein signaling. For this purpose, we analyzed the effect of tungstate on heterologously expressed BKαβ_1_ channels in the presence of GDPβS (500 μM) that locked the Gα protein subunit in its inactive (GDP-bound) state or after pre-incubation of the transfected cells with PTX (500 ng/ml). Fig. 2 (A, C) shows representative BKαβ_1_ currents, recorded before (control) and after the addition of 1 mM tungstate (WO_4_^2-^) to a nominally Ca^2+^-free bath (intracellular) solution containing 0.7 mM Mg^2+^ (an intracellular cation required for the tungstate-induced activation of BK channels [14]). Changes in BKαβ_1_ channel activity were determined by plotting the G-V relationships of the measured BK tail currents, before and after exposure to tungstate (Fig. 2B, 2D). For each G-V curve, the voltage for channel half-activation (V_1/2 act_) was calculated (Fig. 2E). We found that even in the presence of G-protein inhibitors, tungstate still decreased by ~16–25 mV the V_1/2 act_ for BKαβ_1_ channels (P < 0.01, paired t-test), favouring channel activation by voltage. This action of tungstate on BKαβ_1_ channels was similar to the one we have previously reported (a decrease of V_1/2 act_ by ~22 mV), under identical experimental conditions but without interfering with the activation of G proteins [14]. Thus, these results ruled out an involvement of G proteins in the tungstate-induced activation of BKαβ_1_ channels. Instead, the data suggested that the BK-mediated and G_i/_o-dependent phosphorylation of ERK1/2 induced by tungstate involved the activation of G_i/o_ proteins downstream of tungstate interaction with the BK channel. To further test this idea, we evaluated whether G_i_ protein activation by tungstate was related to the presence of BKαβ_1_ channels. G protein activation was evaluated by measuring the Fluorescence Resonance Energy Transfer (FRET) between α_i_ and β subunits of the heterotrimeric G protein tagged with the yellow fluorescent protein (YFP) and the cyan fluorescent protein (CFP), respectively. It has been reported that G protein subunits undergo a molecular rearrangement during activation (rather than a complete dissociation). Thus, when the CFP was fused to the N-terminus of the Gβ subunit, activation of the G protein following stimulation of G_i_ protein-coupled adrenergic α_2A_ receptors (α_2A_-ARs) with noradrenaline (NA) resulted in an increase in FRET (measured as an elevation of the ratio between YPF and CFP fluorescence emission (F_YFP_/F_CFP_)) [40].

**Fig 3:**
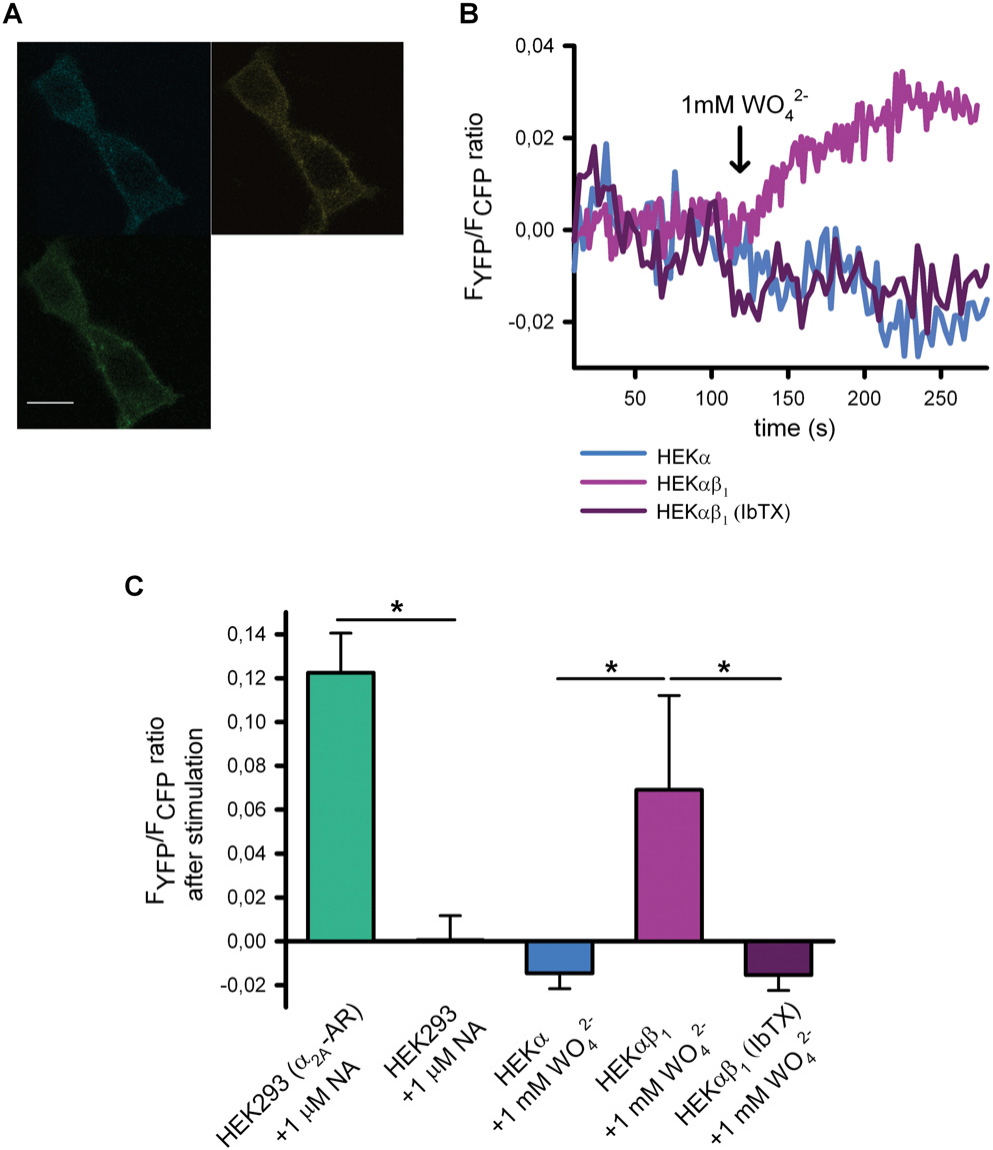
Tungstate-induced activation of heterologously expressed Gi/o proteins is mediated by BKαβ_1_ channels. (A) Example of the reinforced membrane fluorescence pattern and emission levels from CFP channel (up-left), YFP (FRET channel) (up-right) and the merge channels (bottom-left) from HEK293 heterologously expressing Gα_i_-YFP and CFP-Gβ. Fluorescence microscopy images were recorded by using confocal microscopy (for more details see Methods). FRET signal was determined by using donor ratiometric parameters (458/514) after excitation in the CFP frequency and registering in the YFP emission frequency. (B) Representative FRET changes from HEK293, HEKα or HEKαβ_1_ cells transfected with the cDNAs of G protein subunits, in response to 1 mM tungstate (either in the absence or presence of IbTX), as indicated. (C), Average FRET changes for the different experimental conditions illustrated in B (n = 5–9). *P < 0.05 (Kruskal-Wallis test followed by Dunn *post hoc* test).

Gα_i_-YFP and CFP-Gβ subunits (along with the Gγ subunit) were heterologously expressed in HEKα and HEKαβ_1_ cells and FRET measurements were carried out before and after the addition of tungstate. Changes of FRET in response to noradrenaline (NA) in HEK293 cells co-expressing the above mentioned G protein subunit constructs along with α_2A_-ARs, were used as positive control. Cells with a reinforced membrane fluorescence pattern (Fig. 3A), indicating the co-localization of the expressed fluorescent G protein subunits in the cellular membrane, were selected for FRET measurements. Addition of tungstate (1 mM) only increased FRET in HEKαβ_1_ but not in HEKα cells expressing Gβ_i_-YFP and CFP-Gβ subunits (Fig. 3B (magenta and blue traces, respectively) and 3C). As previously reported [40], addition of NA (1 μM) to HEK293 cells co-transfected with the cDNAs of G protein subunits and the α_2A_-AR, resulted in an increase in FRET (elevated F_YFP_/F_CFP_ ratio) (Fig. 3C). No increase in FRET after addition of NA was seen when the cDNA of the α_2A_-AR was omitted in the cell transfection process (Fig. 3C). The tungstate-induced elevation of the FRET signal in HEKαβ cells was about 56% of that produced by NA in HEK293 cells co-expressing the α_2A_-AR (Fig. 3C). Furthermore, as observed for the enhanced tungstate-induced phosphorylation of ERK found in HEKαβ_1_ cells (Fig. 1E, 1F), the increase in FRET among G protein subunits after tungstate application was abolished by pre-incubating the HEKαβ_1_ cells with IbTX (Fig. 3B (dark purple trace) and 3C). Together, these data suggested that tungstate-targeting of the BKαβ_1_ channel promoted G_i_ protein activation.

### Tungstate effect on Ca^2+^-activated K^+^ channels is specific for BK channels

We evaluated whether the tungstate effect on BK channel activity applied also to other distantly related Ca^2+^/calmodulin-gated K^+^ channels, in particular the K_Ca_3.1 (IK1) channel that has been previously related to the direct activation of the ERK signaling pathway. S1 Fig. shows that pre-activated (by 1 μM intracellular Ca^2+^) K_Ca_3.1 currents recorded from murine 3T3 fibroblasts were insensitive to 1 mM tungstate, even after current potentiation by the K_Ca_3.1 channel activator SKA-31 [41].

### Tungstate-induced activation of native BK channels in vascular smooth muscle cells depends on the expression of the regulatory BK channel β_1_ subunit

**Fig 4:**
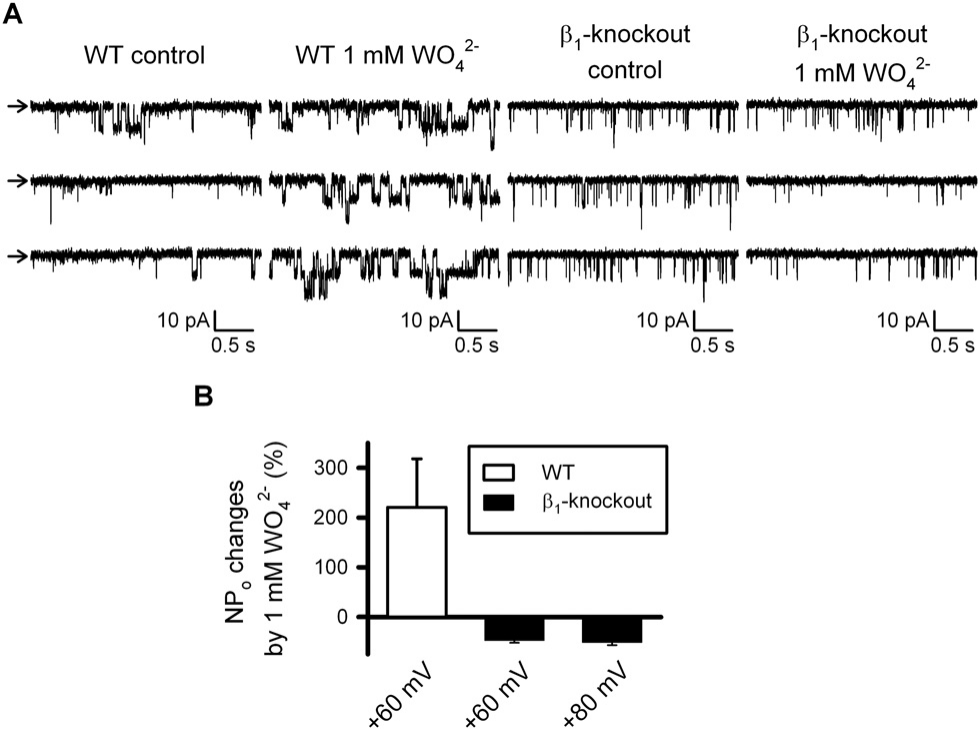
Tungstate-induced increase in the open probability of BK channels endogenously expressed in freshly isolated vascular myocytes requires the BK channel β_1_ subunit. (A) Representative recordings obtained from inside-out patches clamped at + 60mV from wild-type (WT) and β_1_- knockout freshly isolated mouse vascular myocytes, before (control) and after exposure to 1 mM tungstate (1 mM WO_4_^2-^), as stated. Arrows indicates the closed state level. (B) Average changes (in %) of BK channel open probability (NP_o_) induced by 1 mM tungstate on WT and β_1_- knockout mouse vascular myocytes at the indicated voltage membrane. Significant differences were found among WT (n = 7) and β_1_- knockout (n = 9), P < 0.001 (Student’s t-test). Further depolarization to +80 mV of β_1_- knockout inside-out patches did not change the response to tungstate found at +60 mV.

We have previously reported that the positive action of tungstate on BKαβ_1_ channel activity, found in a heterologous expression system, was likely to be responsible of the tungstate-induced vasodilatation of mouse arteries pre-contracted with endothelin-1. This conclusion was based on two experimental evidences: tungstate-induced vasorelaxation depended on the expression of the regulatory β_1_ subunit (vasorelaxation was lost in β_1_-knockout arterial rings) and it was not related to the reported tungstate-induced inhibition of the endothelial xanthine oxidase (XO) [8] as it was not replicated by the XO blocker allopurinol [14]. We have now studied directly the effect of tungstate on BK channels endogenously expressed in murine vascular smooth muscle cells (VSMCs). Single-channel recordings showed that BK channel open probability (NP_o_) increased in response to 1 mM tungstate only in inside-out patches obtained from VSMCs of wild-type mice but not in those from β_1_- knockout mouse VSMCs (Fig. 4).

### Tungstate promotes ERK1/2 phosphorylation and reduces PDGF-stimulated cell proliferation in human vascular smooth muscle in a BK channel-dependent manner

**Fig 5:**
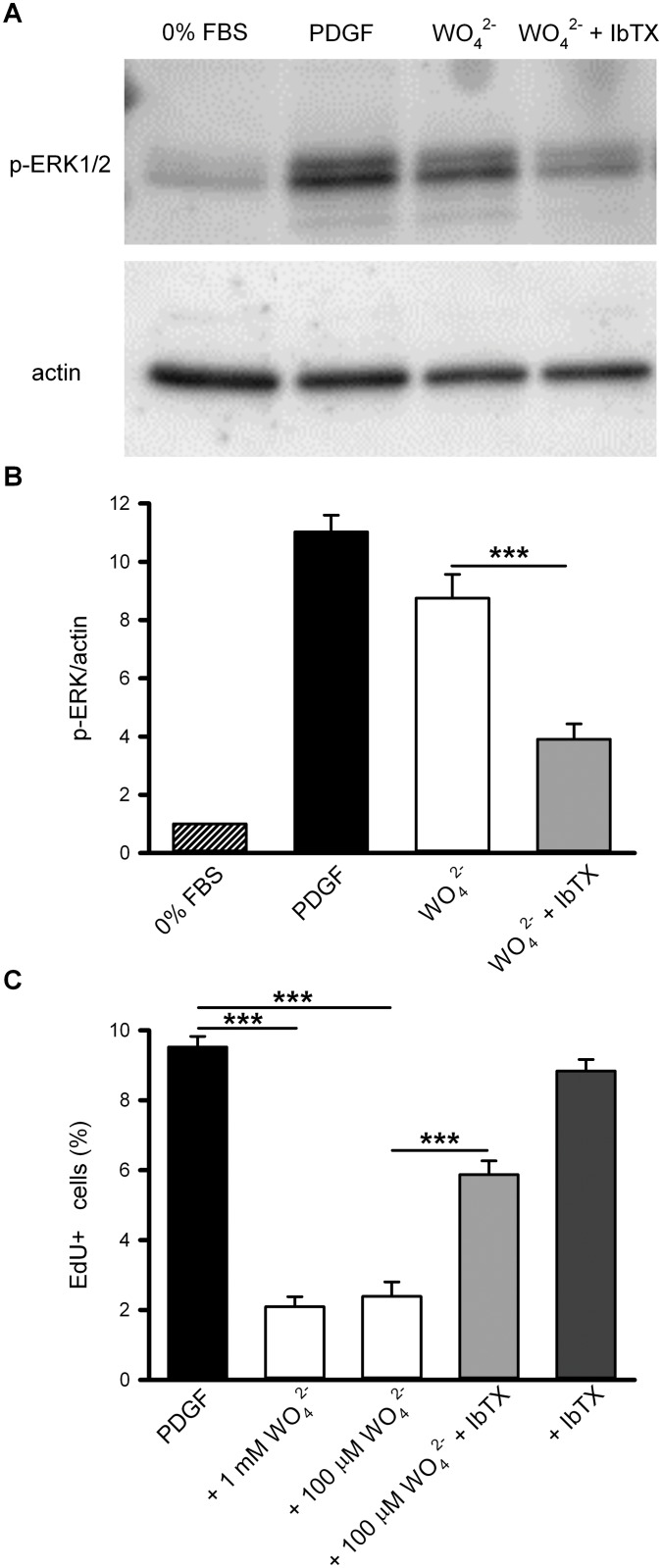
BK channels mediate both ERK1/2 phosphorylation and reduction of PDGF-stimulated proliferation induced by tungstate in human vascular smooth muscle cells. (A) Representative Western-blot showing phosphorylation of ERK1/2 using phospho-ERK-specific antibodies after 10 minutes incubation in control conditions (0% FBS), with PDGF (20 ng/ml), and with tungstate (1 mM) alone or in combination with iberiotoxin (IbTX 100 nM), as indicated. Beta-actin was used as loading control. (B) Protein expression and phosphorylation were quantified by densitometry of the corresponding Western blot signal and normalized to beta-actin. The ratio p-ERK/beta-actin in control conditions was taken as 1, so that fold-changes relative to control are shown. (C) VSMCs were serum starved for 48 hours and then incubated 30 hours with 20 ng/ml PDGF alone or in combination with the indicated compounds. During the last 6 hours of incubation, EdU was added to the media to detect the number of cells entering S-phase. Mean ± S.E.M. of 6–9 determinations from at least four different cultures. ***P < 0.001 (ANOVA followed by Tukey *post hoc* test).

We next analyzed whether tungstate also promoted ERK phosphorylation in human VSMCs and its relationship with the BK channel. Similar to that seen in HEKαβ_1_ cells (constitutively expressing the bovine α and β_1_ subunits of the BK channel), treatment of human VSMCs with tungstate (1 mM for 10 minutes) promoted the phosphorylation of ERK1/2 up to slightly lower levels than PDGF treatment *although this difference did not reach statistical significance* (P > 0.05, ANOVA followed by Tukey *post hoc* test). Such tungstate-induced ERK phosphorylation in human VSMCs was significantly reduced by pretreatment with the specific BK channel blocker iberiotoxin (IbTX, 100 nM) (Fig. 5A and 5B). As Gβγ-stimulated ERK1/2 phosphorylation has been reported to lead to differentiation of VSMCs to a contractile phenotype [36, 37], we also studied the effect of tungstate on PDGF-induced human VSMC proliferation. For this analysis, we lowered tungstate to 100 μM because we have previously reported that at this lower concentration, tungstate still promoted voltage-dependent activation of BKαβ_1_ channels and induced vasorelaxation in endothelin-1-pre-contracted mouse arteries [14]. Besides, such tungstate concentration is only slightly higher than the tungstate levels measured in the plasma of both experimentally treated rodent models and humans (~5–20 μM) [2, 42]. We found that 100 μM tungstate strongly reduced the proliferative action of PDGF (the effect was similar when using 1 mM tungstate). IbTX prevented significantly this inhibition. IbTX alone had no significant effect on PDGF-induced VSMC proliferation (Fig. 5C).

## Discussion

Tungstate is a transition metal that exerts antidiabetic [2, 43], antiobesity [6] and antihypertensive actions [8–10] in several experimental animal models. Despite considerable knowledge on the pharmacological and metabolic effects of tungstate, little information exists regarding its molecular mechanisms of action. In this sense, it is known that tungstate triggers intracellular signaling pathways related to the activation of extracellular signal-regulated kinases (ERK) in several cell types [4, 11, 12]. This signaling action of tungstate mimics the effect of insulin in hepatocytes, by increasing glycogen deposition but in an insulin receptor-independent manner. Tungstate activates PTX-sensitive G_i_ proteins that in turn activate the small GTPase Ras to produce the phosphorylation of ERK, the subsequent phosphorylation of p90rsk and glycogen synthase kinase-3β, and the activation of glycogen synthase leading to glycogen deposition [4, 13]. The antihypertensive effect of tungstate has been linked to both the inhibition of the endothelial xanthine oxidase [8] and the activation of the large conductance voltage- and Ca^2+^-activated K^+^ (BK) channel at the vascular smooth muscle cells (VSMCs) [14]. Vascular BK channels are formed by the pore-forming α (KCNMA1) and the regulatory β_1_ (KCNMB1) subunits. Indeed, we reported previously that tungstate only activates BK channels containing either the β_1_ or the β_4_ (but no β_2_ or β_3_) subunits [14, 26].

**Fig 6:**
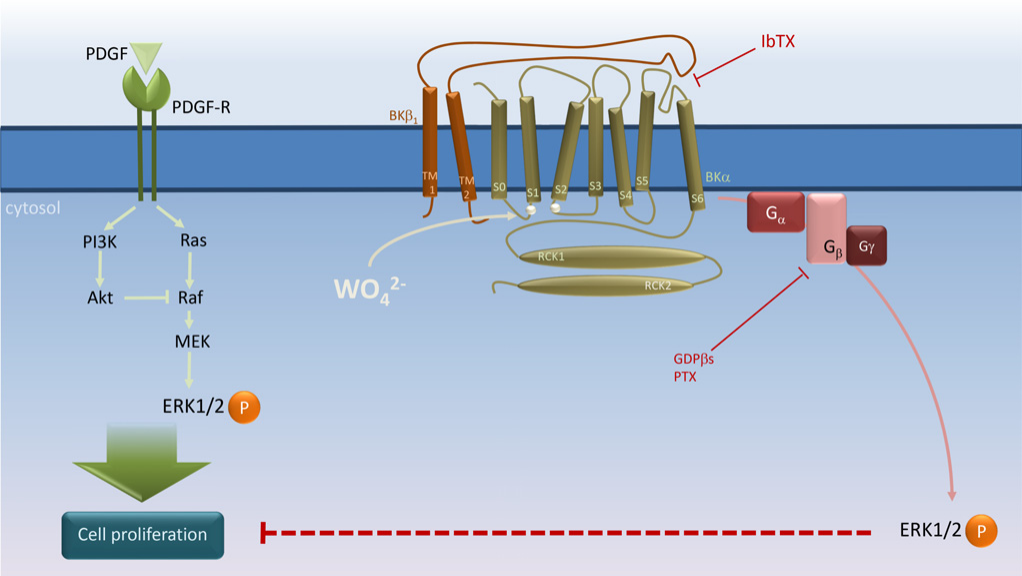
Schematic representation of BKαβ_1_ channel’s role in tungstate-induced, G_i_ protein-mediated ERK phosphorylation and its interaction with PDGF-stimulated cell proliferation in human vascular smooth muscle. PDGF stimulates both the Ras/Raf/MEK/ERK and the phosphatidylinositol 3-kinase (PI3K)/Akt mitogenic signaling pathways to induce vascular smooth muscle cell (VSMC) proliferation [35]. Tungstate (WO_4_^2-^) binding to the BK channel α subunit at a site involving residues of the Voltage Sensor Domain (silver circles in the S0–S1 and S2–S3 linkers) [14, 26] promotes activation of the voltage sensor and channel gating in a β_1_ subunit-dependent manner, and subsequently, activation of PTX-sensitive G proteins to induce ERK phosphorylation and inhibition of PDGF-stimulated VSMC proliferation. Binding of iberiotoxin (IbTX) to the external mouth of the channel in close proximity to the β_1_ extracellular loop, which plays an important role in the modulation of voltage sensor activation and gating of the BK channel [21, 46–53], may impair the conformational changes in the BKαβ_1_ channel produced by tungstate to prevent the coupling of channel gating to G protein activation without the need of K^+^ conduction. For further details, please see Discussion.

Here, we provide evidence that tungstate-targeting of β_1_ subunit-containing BK channels promotes the activation of PTX-sensitive G_i_ proteins to enhance the tungstate-induced phosphorylation of ERK (Fig. 6). First, we observed significant higher levels (~40–44%) of ERK phosphorylation after tungstate treatment in HEK293 cells expressing both BK channel α and β_1_ subunits (HEKαβ_1_ cells) than in cells that did not express BK channels (HEK293 cells) or expressed the BK pore-forming α subunit alone (HEKα cells). Second, the fact that such enhancement of the tungstate-induced activation of the ERK pathway found in HEKαβ_1_ cells is prevented by either PTX or IbTX, supports the involvement of both G_i/o_ proteins and BK channels in this signaling process. Third, G_i/o_ proteins are not upstream of the tungstate-induced activation of BKαβ_1_ channels, since tungstate-induced changes in BK channel activity remains unaltered even in the presence of the G protein inhibitors PTX or GDPβS. This observation is in agreement with previous electrophysiological data and comparative structural analysis suggesting that tungstate modulates BK channel activity by direct binding to a site located at the BK α subunit (around residues of the voltage sensor and the RCK1 domains that coordinate the binding of Mg^2+^) [14], with the required participation of β_1_ subunit extracellular loop residues that stabilize the active configuration of BK channel voltage sensor [26]. And fourth, BKαβ_1_ channels seem to be upstream in the tungstate-induced, G_i/o_ protein-mediated ERK phosphorylation pathway. Thus, tungstate only activated heterologously expressed G_i_ proteins (indicated by an increase in FRET among Gα_i_-YFP and CFP-Gβ subunits) in HEKαβ_1_ cells, but no in HEKα cells, an effect that was prevented by the blockade of BKαβ_1_ channels with IbTX. Altogether, these results suggest that BKαβ_1_ channels might well act as tungstate receptors to trigger the activation of the ERK pathway. Among Ca^2+^-activated K^+^ channels, such tungstate receptor function seems to be specific for BKαβ_1_ channels: we observed that the IK1 channel, which has been related to the direct activation of the ERK signaling pathway [30], is tungstate-insensitive.

Given that the BK β_1_ channel subunit is primarily found in VSMCs, where it improves channel function for a better fine-tuned and efficient negative feedback on vascular tone [19, 20], the functional relationship between BKβ_1_ channels and the G_i/o_ protein-ERK signaling cascade might also have physiological and/or pathological relevance in the vascular beds. It is well established that phenotypic remodeling of VSMCs, involving changes from contractile toward a more proliferative phenotype caused by mitogen-stimulated gene expression, occurs during the pathogenesis of hypertension, atherosclerosis or vascular restenosis [31, 32]. Therefore, treatments that oppose such remodeling and promote VSMC re-differentiation can be of help in the management of pathological vascular remodeling. Growth factors such as platelet-derived growth factor (PDGF), by acting on specific tyrosine kinase receptors, stimulate several mitogenic signaling pathways that lead to VSMC proliferation: (i) phospholipase C (PLC) isoforms that increase the cytosolic Ca^2+^ levels and activate protein kinase C, (ii) the Ras/Raf/MEK/ERK pathway, and (iii) the phosphatidylinositol (PI) 3-kinase/Akt pathway [35]. These same intracellular signaling routes promote VSMC differentiation in response to serum components such as thrombin, acting via G-protein coupled receptors and G_i/o_ βγ dimmers [35–37]. The opposite effects produced by these two kinds of agonists regarding the modulation of VSMC phenotype seem to be due to differences in both the intensity of the signals and their kinetic patterns, along with the cross-talk between pathways [35]. Thus, upon stimulation of tyrosine kinase receptors there is a rapid and strong phase of ERK phosphorylation and a robust PI 3-kinase-dependent and sustained Akt phosphorylation, which prevents the generation of a late ERK activation phase by inhibiting Raf kinase activity and suppressing MEK (Fig. 6) [35]. Interestingly, it has been reported that growth factor-dependent mitogenesis relies on two distinct signaling phases. The first phase involves MEK/ERK and c-Myc activation to make cells go through the initial phase of the G0 to S cell cycle interval. Such first phase is required to engage the components necessary for cell cycle progression during a second phase that depends exclusively on PI 3-kinase lipid products [44]. However, after agonist binding to GPCR and G protein activation, G βγinduces not only the rapid and strong phase of ERK phosphorylation but also a late phase that is required for changes in gene expression leading to VSMC differentiation. Such second phase of ERK activation can occur because, in this case, Akt phosphorylation is weak and transient and fails to inhibit Raf kinase activity [35].

Here we show that the open probability of endogenous BK channels in murine VSMCs is increased by tungstate in a β_1_-dependent manner. This result is consistent with our previous data showing that tungstate activates BK channels containing either the β_1_ or the β_4_ (but not β_2_ or β_3_) subunits in a heterologous expression system, and that tungstate-induced relaxation of mouse pre-contracted blood vessels depends on the expression of the regulatory BK β_1_ subunit [14]. Functional BK channels have been also recorded from the cultured human renal VSMCs used in this study (data not shown). In addition, expression studies in proliferating VSMCs show that they retain expression of both BKα and BKβ_1_ mRNA, presenting an increase of BKαβ_4_ mRNA [45]. We have now found that targeting of vascular BK channels by tungstate also promotes phosphorylation of ERK in human renal VSMCs. Furthermore, tungstate inhibits the PDGF-stimulated human VSMC proliferation, in a process that is also mediated, at least in part, by BK channels (Fig. 6). However, further research is required in order to establish whether the activation of the G_i/o_ protein/ERK pathway lies behind such antiproliferative action of tungstate, by allowing the late phase of ERK phosphorylation. In this sense, it has been shown that abrogation of the PI 3-kinase/Akt signaling changes the PDGF-induced proliferative VSMC phenotype toward enhanced expression of contractile proteins [35].

The question of how the targeting of BKαβ_1_ channels by tungstate leads to the activation of G_i/o_ proteins (and the subsequent ERK phosphorylation and inhibition of VSMC proliferation stimulated by PDGF), needs to be answered by future studies. Nonetheless, the fact that this signaling process is abolished in the presence of the BK channel blocker IbTX, might initially suggest that the conduction of K^+^ ions through the tungstate-activated channel is involved in G protein activation. IbTX shares a high sequence identity (around 68%) with charybdotoxin (ChTX) and a similar mechanism for BK channel blockade has been suggested [46]. The exact interaction site for the toxins in BK channels is not clear but the involvement of residues around the outer mouth pore of the channel has been proposed [47]. In agreement, the crystal structure of a mutant form of the voltage-gated K^+^ channel K_V_1.2 in complex with ChTX indicates that the toxin binds to the extracellular pore entryway and interacts directly with K^+^ inside the selectivity filter [48]. Furthermore, some residues in the extracellular loop of the BK regulatory β_1_ subunit are responsible of the high BK channel affinity for ChTX [21, 49]. These β_1_ loop amino acids are in close proximity to the external mouth and, perhaps, the selectivity filter and gate of the channel [49–51]. Besides, different studies indicate that the β_1_ extracellular loop plays an important role in the modulation of voltage sensor activation and gating of the BK channel [52, 53]. Therefore, toxin binding to the BKαβ_1_ channel may also modify the structural changes related with the activation of the voltage sensor and channel gating. Then, toxin impairment of the conformational changes in the BKαβ_1_ channel produced by tungstate may affect the coupling of channel gating to G protein activation without the need of K^+^ conduction (Fig. 6). In support of this hypothesis, we have found that two distinct concentrations of tungstate (100 μM and 1 mM), which increase and reduce (respectively) K^+^ flux through vascular BKαβ_1_ channels, equally inhibit PDGF-induced cell proliferation in human vascular smooth muscle (Fig. 5C). Indeed, 1 mM tungstate has a dual effect on heterologously expressed BKαβ_1_ channels: it reduces (by ~ 30–40%) the amplitude of K^+^ currents (an effect that is equal for BK channels containing or not the different regulatory β subunits, β_1_-β_4_) and favors channel activation by voltage (i.e. decreases V_1/2 act_ by ~ 20 mV) in a Mg^2+^- and β_1_ subunit-dependent manner [14]. From previous studies of tungstate effects on murine arterial contractility, and given the key role of BKαβ_1_ channels in this process [18–20], it can be inferred that the negative action of 1 mM tungstate on vascular BK current amplitude outweighs its positive effect on the voltage-dependent activation of the channel. Thus, a net reduction of vascular BK currents can explain the constriction of arterial rings from both wild-type (WT) and β_1_-knockout mice induced by 1 mM tungstate [14]. On the contrary, at micromolar levels (100 μM) tungstate still promotes voltage-dependent activation of the vascular (β_1_ subunit-containing) BK channel without reducing K^+^ current amplitude. The resulting net increase in the activity of vascular BKαβ_1_ channels explains why 100 μM tungstate only promotes relaxation of WT but not β_1_-knockout mouse arteries pre-contracted with endothelin-1 [14].

In the same line of thought, it has been shown that activation of the ERK signaling pathway mediated by IK1 channels does not require the capability of this channel to conduct K^+^ ions [30]. There are evidences that support the existence of a direct protein-protein crosstalk among BK channels and some G-protein coupled receptors, such as μ-opioid [54] or thromboxane A2 receptors [55]. In addition, it has been shown that BK α channel subunit can directly interact with Gβγ dimmers [56].

In summary, our data provide evidence to consider BK channels as another member of the growing list of channels directly involved in the transduction of intracellular signals, beyond their ion-conducting function [29, 30]. Our results further highlight the relevance of BK channel function in VSMCs, apart from its well known role in reducing cell contractility to promote vasorelaxation, arising also as a potential target for the modulation of the growth factor-induced proliferative phenotype associated with certain vascular pathologies. In this sense, the functional interaction between tungstate and vascular BKαβ_1_ channels become an interesting starting point to develop new antihypertensive therapies [14, 57].

## Supporting Information

S1 Fig Tungstate has no effect on K_Ca_3.1 (IK1) channel activity. (A) Whole-cell recordings of K_Ca_3.1 in murine 3T3 fibroblasts. K_Ca_3.1 currents were pre-activated by infusion of 1 μM Ca^2+^ via the patch-pipette as previously described [38]. Pre-activated currents (control) were not changed by 1 mM tungstate (1 mM WO_4_^2-^). (B) Average data showing pre-activated K_Ca_3.1 current densities before and after tungstate application (P > 0.99, Mann-Whitney U-test; n = 4). (C) Average changes (in %) of pre-activated K_Ca_ 3.1 currents in response to 1 mM tungstate (1 mM WO_4_^2-^) in the absence or presence of K_Ca_3.1 activator SKA-31, as indicated. The SKA-31-potentiated K_Ca_3.1 current was likewise insensitive to 1 mM tungstate (P = 0.56, Mann-Whitney U-test). Numbers in brackets indicate the number of cells tested in each experimental condition.
(DOCX)
