# Supplementary material for: Tungstate-Targeting of BKαβ1 Channels Tunes ERK Phosphorylation and Cell Proliferation in Human Vascular Smooth Muscle
Source: PLoS One. 2015 Feb 6;10(2):e0118148. doi: 10.1371/journal.pone.0118148 (PMC4320054; doi:10.1371/journal.pone.0118148)
Supplement: S1 Fig — (A) Whole-cell recordings of KCa3.1 in murine 3T3 fibroblasts. KCa3.1 currents were pre-activated by infusion of 1 μM Ca2+ via the patch-pipette as previously described [38]. Pre-activated currents (control) were not changed by 1 mM tungstate (1 mM WO4 2-). (B) Average data showing pre-activated KCa3.1 current densities before and after tungstate application (P > 0.99, Mann-Whitney U-test; n = 4). (C) Average changes (in %) of pre-activated KCa 3.1 currents in response to 1 mM tungstate (1 mM WO4 2-) in the absence or presence of KCa3.1 activator SKA-31, as indicated. The SKA-31-potentiated KCa3.1 current was likewise insensitive to 1 mM tungstate (P = 0.56, Mann-Whitney U-test). Numbers in brackets indicate the number of cells tested in each experimental condition. (DOCX) [file pone.0118148.s001.docx]

**
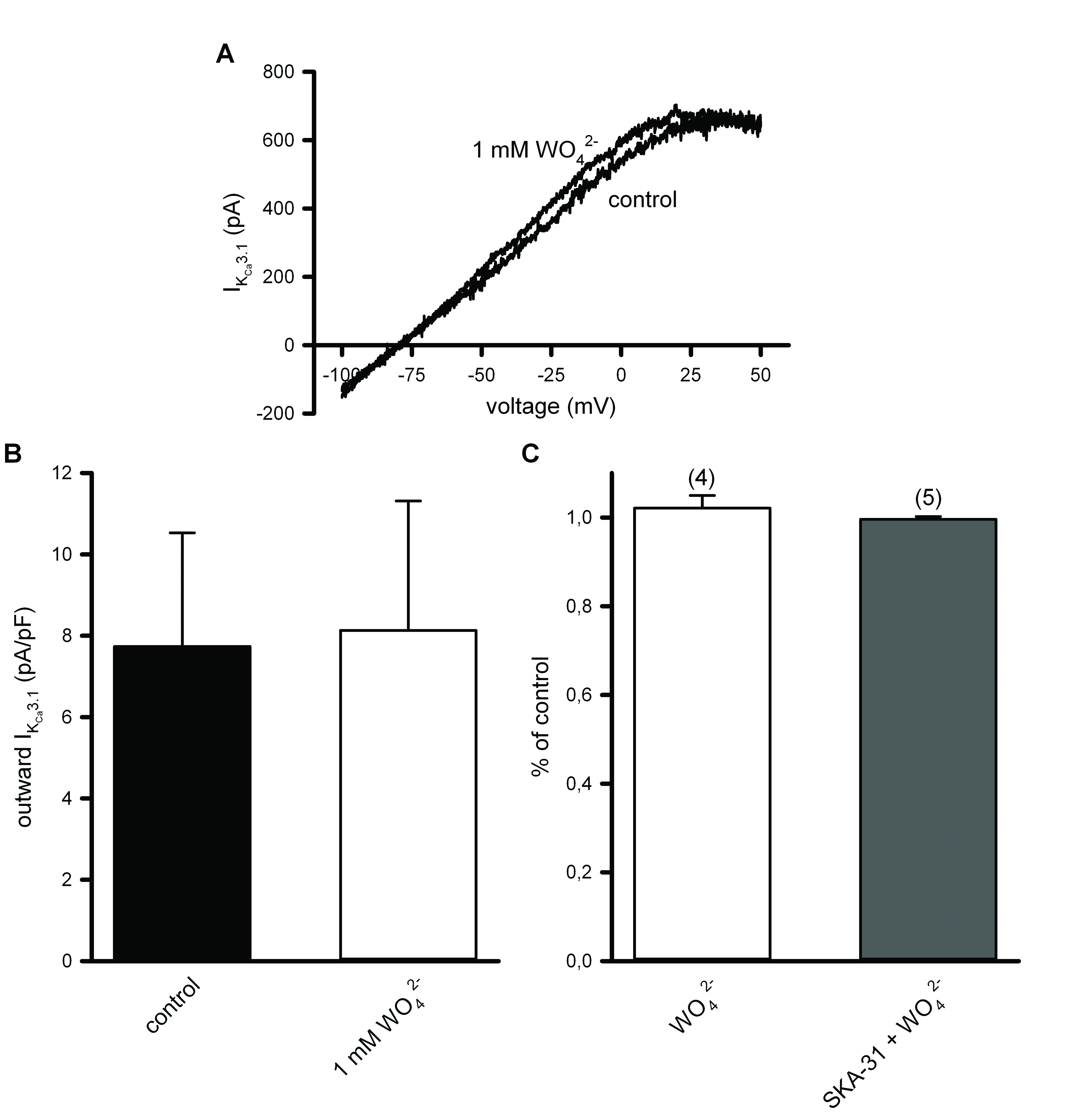
Supporting Information**

**Figure S1**

**Figure S1. Tungstate has no effect on K_Ca_3.1 (IK1) channel activity.** (A) Whole-cell recordings of K_Ca_3.1 in murine 3T3 fibroblasts. K_Ca_3.1 currents were pre-activated by infusion of 1 μM Ca^2+^ via the patch-pipette as previously described [38]. Pre-activated currents (control) were not changed by 1 mM tungstate (1 mM WO_4_^2-^). (B) Average data showing pre-activated K_Ca_3.1 current densities before and after tungstate application (P > 0.99, Mann-Whitney U-test; n = 4). (C) Average changes (in %) of pre-activated K_Ca_ 3.1 currents in response to 1 mM tungstate (1 mM WO_4_^2-^) in the absence or presence of K_Ca_3.1 activator SKA-31, as indicated. The SKA-31-potentiated K_Ca_3.1 current was likewise insensitive to 1 mM tungstate (P = 0.56, Mann-Whitney U-test). Numbers in brackets indicate the number of cells tested in each experimental condition.
